# 单操作孔肺叶切除术治疗周围型肺癌的临床研究

**DOI:** 10.3779/j.issn.1009-3419.2013.09.09

**Published:** 2013-09-20

**Authors:** 畅 李, 海涛 马, 靖康 何, 斌 倪, 春 徐, 军 赵

**Affiliations:** 215006 苏州，苏州大学附属第一医院心胸外科 Department of Toracic and Cardiovascular Surgery, the First Afliated Hospital of Soochow University, Suzhou, 215006, China

**Keywords:** 单操作孔, 肺叶切除术, 肺肿瘤, Single utility port, Lobectomy, Lung neoplasms

## Abstract

**背景与目的:**

胸腔镜肺叶切除术在肺癌治疗领域的价值与可行性已被广泛接受。本研究探讨单操作孔肺叶切除术治疗周围型肺癌的临床效果。

**方法:**

回顾性分析2011年2月-2013年1月苏州大学附属第一医院心胸外科经单操作孔胸腔镜肺叶切除术治疗的周围型肺癌患者87例（单操作孔组），用同期经传统“三孔法”肺叶切除术治疗的周围型肺癌作为对照（“三孔法”组）。比较两组患者的手术时间、术后下床活动时间及住院时间、术中出血量、术后总引流量、术后拔管时间、清扫淋巴结数量及术后并发症、术后疼痛的发生情况。

**结果:**

两组患者手术过程顺利，单操作孔组与三孔法组手术时间（151.03±25.97）min *vs* (156.27±26.49）min、淋巴结清扫数目（13.06±1.36）枚*vs*（12.61±1.56）枚、术中出血量（188.62±47.03）mL *vs*（179.60±28.96）mL及术后并发症发生率（18/87）*vs*（21/75）差异无统计学意义（*P* > 0.05)；术后下床活动时间（11.17±8.69）*vs*（13.76±7.43）h、术后住院天数（7.18±1.95）天*vs*（7.92±2.03）天、拔除引流管时间（3.85±1.21）天*vs*（4.43±1.43）天及总引流量（671.49±178.31）mL *vs*（736.93±170.39）mL的差异有统计学意义（*P* < 0.05），两组患者术后视觉模拟评分法（vision analogue score, VAS）评分变化差异有统计学意义（*P* < 0.01）。

**结论:**

单操作孔肺叶切除术治疗周围型肺癌可以达到与传统“三孔法”全腔镜肺叶切除术相同的效果，是一种有发展前途的手术方式。

## Abstract (en)

**Background and objective:**

Video-assisted thoracoscopic surgery (VATS) lobectomy is now generally accepted for patients with lung cancer. The aim of this study is to review the technology of thoracoscopic lobectomy with single utility port in the treatment of peripheral lung cancer.

**Methods:**

We retrospectively analyzed the clinical data of 87 patients with peripheral lung cancer who underwent single utility port complete VATS lobectomy from February 2011 to January 2013 in the First Affiliated Hospital of Soochow University (single utility port group), and compared them with 75 patients with peripheral lung cancer who underwent conventional, 3-port VATS lobectomy in the same period (3-port group). The clinical outcomes including operation time, time to first activity out of bed, postoperative hospital stay, intraoperative blood loss, postoperative drainage volume, chest drainage duration, lymph node dissection number, postoperative complications and degree of chest pain were compared between the two groups.

**Results:**

No perioperative death was observed in both groups. There was no statistical difference in operation time (151.03±25.97 min *vs* 156.27±26.49 min), lymph node dissection number (13.06±1.36 *vs* 12.61±1.56), intraoperative blood loss (188.62±47.03 mL *vs* 179.60±28.96 mL) and incidence of serious postoperative complications (18/87 *vs* 21/75) between the two groups. There were statistical differences in time to first activity out of bed (11.17±8.69 h *vs* 13.76±7.43 h), postoperative hospital stay (7.18±1.95 d *vs* 7.92±2.03 d), chest drainage duration (3.85±1.21 d *vs* 4.43±1.43 d) and total postoperative drainage volume (671.49±178.31 mL *vs* 736.93±170.39 mL) between the two groups (*P* < 0.05). The change of vision analogue score (VAS) score between the two groups after operation was also statistically significant (*P* < 0.01).

**Conclusion:**

The completely thoracoscopic lobectomy with single utility port is a safe and feasible surgical procedure compared with conventional 3-port VATS lobectomy for selected patients.

近年来随着胸腔镜外科（video-assisted thoracoscopic surgery, VATS）的发展，越来越多的单位开始将这一技术应用于临床，从早期的肺大疱切除、肺活检术及简单纵隔肿瘤切除术，逐渐发展到包括肺癌根治术、食管癌切除术等几乎各种胸外科手术^[1]^。在肺癌治疗领域，胸腔镜肺叶切除术的价值与可行性已被广泛接受^[2-4]^，早于2006年、2007年已成为美国国家综合癌症网络（National Comprehensive Cancer Network, NCCN）和美国胸科医师协会（American College of Chest Physicians, ACCP）肺癌治疗指南中肺癌的标准手术方式。目前国内外应用最多的胸腔镜肺叶切除术一般为“三孔法”^[5, 6]^，即包括观察孔、主操作孔和一个副操作孔，由助手经副操作孔进行牵拉、暴露，术者经主操作孔完成分离、结扎等操作。这一术式经多年发展已臻成熟，多项临床研究^[2-4]^均证实该术式应用于经过合理选择的患者可达到与传统开胸手术同样的手术效果。但“三孔”手术仍存在一些尚待改良的问题，例如背部切口疼痛，感觉与运动障碍等。苏州大学附属第一医院心胸外科在长期实践基础上，总结国内外经验，对一部分经选择的病例开展了单操作孔胸腔镜肺叶切除术用于治疗周围型肺癌，取得了较好效果。

## 1 资料与方法

### 1.1 病例与分组

回顾性分析苏州大学附属第一医院心胸外科2011年2月-2013年1月经单操作孔胸腔镜肺叶切除术治疗的周围型肺癌患者87例，其中男65例，女22例，年龄41岁-87（63.9±12.1）岁。同期采用传统“三孔法”胸腔镜肺叶切除术治疗的肺癌患者75例，其中男52例，女23例，年龄48岁-85（66.2±8.7）岁。所有患者术前均未行放、化疗。术前均行头颅MRI、腹部B超、骨ECT除外远处转移，心、肺功能检查排除手术禁忌。胸部增强CT显示周围型病灶，肿瘤直径 < 5 cm，无明显增大的肺门及纵隔淋巴结。术前常规行纤维支气管镜检查明确病灶部位，确定叶支气管开口未受肿瘤侵犯。两组患者均行全腔镜下肺叶切除+肺门、纵隔淋巴结清扫； < 3 cm病灶先行局部楔形切除经快速冰冻切片证实肺癌诊断，部分病灶位置靠近肺门或肿块不易行局部切除者直接行肺叶切除术。术后病理分期包括Ⅰ期、Ⅱ期，以及部分术前诊断Ⅱ期，术后证实N2淋巴结转移的IIIa期患者。经统计两组患者性别、年龄、病变部位、病理类型及TNM分期无显著差异（表 1）。

**1:** 单操作孔组与三孔组临床资料比较 Comparision of clinical characteristics between groups

Clinical characteristics	Single utility port group (*n*=87)	3-port group (*n*=75)	*t*/*χ*^2^	*P*
Sex			0.581	0.446
Male	65	52		
Female	22	23		
Age (yr)	63.86±12.10	66.20±8.72	1.423	0.157
Tumor location			5.741	0.219
Left upper	23	16		
Left lower	11	15		
Right upper	32	18		
Right middle	8	8		
Right lower	13	18		
Pathological types			2.011	0.156
Squamous cell carcinoma	39	42		
Adenocarcinoma	48	33		
T grade			1.544	0.462
1	28	31		
2	57	42		
3	2	2		
N grade			0.128	0.938
0	33	30		
1	36	31		
2	18	14		
TNM			0.457	0.796
Ⅰ	31	30		
Ⅱ	38	29		
Ⅲ	18	16		

### 1.2 手术方法

#### 1.2.1 体位及切口

所有患者均经双腔管插管全身麻醉后取健侧卧位，手术床摇成折刀位。单操作孔组于腋后线第8肋间作2 cm切口置入Trocar作为观察孔，于腋前线第5肋间作3 cm切口，置入弹性切口保护套作为唯一操作孔，不置入撑开器。所有操作均由术者经操作孔完成，切除完成后肺叶标本置入取物袋经由操作孔取出。“三孔法”组观察孔设置同单操作孔组，于腋前线第3或4肋间作3 cm切口，置入弹性切口保护套作为主操作孔，于背侧肩胛下区听诊三角处作2 cm切口置入Trocar作为副操作孔。

#### 1.2.2 肺叶切除及淋巴结清扫

两组患者均行肺叶切除及肺门、纵隔淋巴结清扫术。肺门处理顺序一般按照静脉、动脉、支气管完成；部分患者因肺裂发育不全，解剖困难，更改顺序为处理动脉前先处理支气管，即按照静脉、支气管、动脉顺序。支气管、血管处理使用内镜切割缝合器，小的血管分支使用Hem-o-Lock或可吸收血管夹夹闭后切断。单操作孔组绝大多数操作均由术者完成，助手立于患者背侧主要负责扶镜。“三孔法”组助手立于患者背侧，协助术者经由副操作孔主要完成牵引、暴露及吸引等操作。两组患者纵隔淋巴结清扫范围相同，均为右侧第2、3、4、7、8、9组，左侧第5、6、7、8、9组。

#### 1.2.3 观察指标和方法

观察并统计两组患者的手术时间、术后下床活动时间及住院时间；术中出血量及术后总引流量，术后拔管时间；术中清扫纵隔淋巴结数量；术后并发症的发生情况；术后疼痛评价采用视觉模拟评分法（vision analogue score, VAS）：共0分-10分，无痛记为0分，最痛记为10分，由患者盲法划线测量并记录。

### 1.3 统计分析

采用SPSS 20.0软件进行统计分析，计量资料采用*t*检验，计数资料采用χ^2^检验，两组患者术后胸痛的VAS评分比较采用重复测量方差分析，分析前先进行球形检验（Mauchly’s test of sphericity）。*P* < 0.05为差异有统计学意义。

## 2 结果

全部患者无围手术期死亡。单操作孔组患者与“三孔法”组患者相比，手术时间、淋巴结清扫数目、术中出血及术后并发症发生率差异无统计学意义（*P* > 0.05)；术后下床活动时间、术后住院天数、拔除引流管时间及总引流量差异有统计学意义（*P* < 0.05)，见表 2；两组患者术后VAS评分变化差异有统计学意义（*P* < 0.01），见图 1。

**2:** 两组患者围手术期资料比较

Outcome	Single utility port group (*n*=87)	3-port group (*n*=75)	*t*/*χ*^2^	*P*
Operation time (min)	151.03±25.97	156.27±26.49	1.267	0.207
Intraoperative bloodloss (mL)	188.62±47.03	179.60±28.96	1.491	0.138
Postoperative hospital stay (d)	7.18±1.95	7.92±2.03	2.353	0.020
Lymphnode dissection number	13.06±1.36	12.61±1.56	1.918	0.057
Time to first activity out of bed (h)	11.17±8.69	13.76±7.43	2.019	0.045
Chest drainage duration (d)	3.85±1.21	4.43±1.43	2.787	0.006
Postoperative drainage volume (mL)	671.49±178.31	736.93±170.39	2.377	0.019
Postoperative complications	18	21	1.178	0.278
Pulmonary atelectasis	8	6		
Arrhythmia	7	10		
Persistent air leak (> 5 d)	3	5		

**1:**
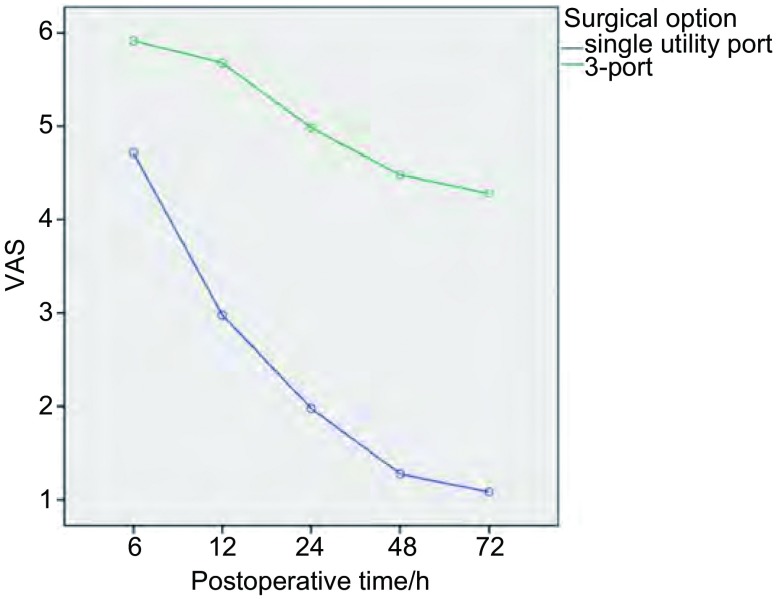
两组患者术后视觉模拟评分法（vision analogue score, VAS）评分的变化 Comparision of postoperative VAS between groups

## 3 讨论

目前外科手术仍是治疗早期非小细胞肺癌的主要方法，常规开胸肺叶切除+系统性纵隔淋巴结清扫术已经被公认为早期肺癌的标准术式。近十年来，随诊微创胸外科技术的进步，VATS肺癌根治术已经在越来越多的单位开展，国内外多篇文献报道VATS肺癌根治术完全可以达到与传统开胸肺癌根治术同样的治疗效果^[2, 3, 7]^，同时创伤小，并发症发生率低，患者恢复更快^[8]^。早期的胸腔镜肺癌根治术往往需借助小切口辅助，逐渐过渡至所有操作均在腔镜下完成的全胸腔镜肺癌根治术，现在国内已有多家单位均已熟练开展该项技术。

在手术切口的选择上，各家报道仍有较多差异。除小切口辅助胸腔镜手术外，“三孔法”是被选择最多的术式，即除观察孔外，于腋前线第3或4肋间及背侧肩胛下区听诊三角处分别做切口作为主、副操作孔。经此路径手术有较多优点，包括显露清楚，方便助手牵拉肺叶、暴露手术部位，术者可以按照与开放手术相同的习惯进行操作等。但“三孔法”也有其不足之处，尤其背部的副操作孔，由于背部肋间隙窄，肌肉层次多，血供丰富，临床实践中常见切口出血；另外由于副操作孔内Trocar反复旋转、压迫肋间神经与上、下肋骨造成术后患者疼痛^[9]^，部分患者甚至因较剧烈的切口疼痛无法下床活动，延长住院时间。针对以上缺点，有学者开始尝试经单一操作孔进行手术，即减少一个副操作孔，全部操作均由术者经主操作孔完成^[10, 11]^，并取得了较好效果。本单位在多年实践基础上，结合国内外经验，完成了本组经单操作孔肺癌根治术病例共计87例，并与同期经传统“三孔法”胸腔镜肺癌根治术比较，效果满意。

全组病例中，绝大多数患者疼痛评分较轻，术后6 h-12 h即可内下床活动，由此减少了术后卧床带来的咳嗽排痰困难、肺不张等并发症风险；而部分“三孔法”组患者由于背部切口疼痛往往需卧床24 h-48 h后方可第一次下床活动，少数患者还需加用杜冷丁等止痛药物。拔除引流管的时间也是影响术后住院时间重要因素，“三孔法”手术中背侧副操作孔所在肋间隙窄，加之术中往往需要反复转动Trocar，对切口周围组织压迫损伤较重，是导致术后引流液增多的主要原因之一。本研究中单操作孔组患者术后总引流量、拔除引流管时间均少于“三孔法”组，而术中出血量、术后并发症发生率与“三孔法”组差别无统计学意义，体现了该术式的优越性。

手术操作方面，我们体会，由于没有副操作孔，所有操作均由术者经前侧主操作孔完成，尤其经常需2-3付器械同时进入，有时会造成相互干扰。克服方法一是使用专用的双关节腔镜器械，可以增大器械在胸腔内的活动度；二是术中通过摇动手术床使肺组织自然向前或向后下垂，帮助显露肺门前后结构；三是通过改良切口设计：观察孔位于腋后线，腔镜进入后正对肺门结构，可以减少对肺组织不必要的牵拉，肺叶切除完成后经此孔观察上纵隔、隆突下等处淋巴结距离最短，视野更为清晰。操作孔位于腋前线第5肋间，各种器械经此孔进入胸腔后与肺裂基本平行，方便使用切割闭合器处理叶裂间动脉及支气管，另外也可避免经第3或第4肋间操作孔进入胸腔后由于上胸腔空间较小，闭合器不易张开等弊端。我们的体会是，使用双关节的腔镜器械经此孔可以顺利完成动脉、支气管处理，切除肺叶后清扫上纵隔、隆突下等处淋巴结时可以做到连带纵隔脂肪组织的整块切除。本组病例中部分分期为N2的患者即属于术前CT未发现明显淋巴结肿大，经系统性淋巴结清扫后分组送病检证实为N2转移。

肺门处理顺序，多数患者仍遵循静脉、动脉、支气管依次完成。由于单孔操作角度受限，必要时需使两孔互换，即经由观察孔进入切割闭合器完成血管或支气管切断；尤其肺静脉几乎常规循此途径完成，可减少反复套带、尝试穿过切割闭合器的时间。部分患者由于肺裂不全，第二肺门解剖较为困难，可以先行处理支气管，从而增加肺门活动度，抵消单孔操作带来的不利影响，但需注意此时牵拉肺组织易因活动度过大而导致肺动脉分支撕裂出血。手术中肺动脉、静脉的游离，打开血管鞘等精细操作主要使用电凝钩，清扫淋巴结等部位则使用超声刀完成，尤其清扫隆突下淋巴结时可以避免电凝损伤支气管动脉导致的出血。本研究统计单操作孔组淋巴结清扫数目与“三孔法”组无统计学差别，说明此法完全可达到肺癌根治术淋巴结清扫的要求；同时手术时间较“三孔法”组并无明显延长。

综上所述，单操作孔胸腔镜肺叶切除术用于治疗周围型肺癌可有效减轻疼痛，缩短恢复时间，同时达到与“三孔法”路径胸腔镜肺癌根治术相当的治疗效果，是一种有前途的微创手术方式，值得推广应用。
